# Enabling mechanistic studies of EVs in vivo: a protocol for isolation and cell-specific labelling in larval zebrafish

**DOI:** 10.1186/s12964-025-02433-3

**Published:** 2025-10-14

**Authors:** Ezgi Kiyga, Katy Reid, Guillaume van Niel, Julie Mazzolini, Dirk Sieger

**Affiliations:** 1 Institute for Neuroscience and Cardiovascular Research, University of Edinburgh, 49 Little France Crescent, Edinburgh, EH16 4SB UK; 2 UK Dementia Research Institute, University of Edinburgh, 49 Little France Crescent, Edinburgh, EH16 4SB UK; 3 CRCI2NA, Nantes Université, INSERM UMR1307, CNRS UMR6075, Université d’Angers, Nantes, France

**Keywords:** Extracellular vesicles (EVs), Tissue digestion, Size exclusion chromatography (SEC), Cell-type specific EV imaging, Zebrafish, In vivo imaging, Development

## Abstract

**Background:**

Extracellular vesicles (EVs) are critical mediators of intercellular communication in development, physiology, and disease. In vivo models such as *Drosophila melanogaster*, *Caenorhabditis elegans*, and *Danio rerio* (zebrafish) now provide powerful platforms to visualize EV dynamics in real time. However, the full potential of these models remains underutilized due to the lack of reliable, cell-specific EV labelling tools and robust EV isolation protocols. Here, we present an optimized workflow for the isolation of EVs from zebrafish larvae and the in vivo labelling of EVs in a cell-type-specific manner.

**Methods:**

To isolate EVs from larval zebrafish, we used size exclusion chromatography (SEC). By comparing different tissue digestion methods and performing step-by-step optimisation of sample preparation prior to SEC, we established a novel protocol that enables EV isolation without compromising cell viability. EV size and concentration were assessed by nanoparticle tracking analysis (NTA), with subsequent characterization by transmission electron microscopy (TEM) and Western blotting. To evaluate the sensitivity of our protocol, we treated zebrafish larvae with GW4869, a known inhibitor of EV biogenesis, and assessed the dose-dependent effects on EV release. To specifically label EVs from distinct cell types, we have generated a UAS: CD63-GFP construct which can be expressed under control of the Gal4 transcriptional activator.

**Results:**

Through a systematic comparison of tissue dissociation techniques, we identify *Bacillus licheniformis* protease as a superior alternative to conventional collagenase treatment, which compromises cell integrity. Treatment with GW4869 confirmed that EV biogenesis and release can be inhibited in a dose-dependent manner and demonstrated that our protocol is sensitive enough to detect and quantify changes in EV levels. To enable cell-specific EV tracking in vivo, we combined the *UAS: CD63-GFP* construct with a radial glia-specific *Gal4* driver line, providing a proof-of-concept for targeted EV imaging in intact tissues.

**Conclusions:**

These advances provide a versatile toolkit for mechanistic studies of EV function in vivo. The broad availability of cell-type-specific Gal4 driver lines in zebrafish and *Drosophila* will now allow researchers to trace EV dynamics from virtually any cell type, while our isolation protocol enables rigorous, quantitative EV analyses across developmental and pathological contexts.

**Supplementary Information:**

The online version contains supplementary material available at 10.1186/s12964-025-02433-3.

## Background

Extracellular vesicles (EVs), particularly small EVs (30–150 nm), are membrane-bound particles released by cells into the extracellular space. These vesicles are present in various biological fluids such as blood, urine, saliva, and cerebrospinal fluid, and play crucial roles in intercellular communication [1–3]. EVs have gathered significant interest in recent years due to their involvement in a variety of physiological and pathological processes, including immune responses, cancer progression, and neurodegenerative diseases [2, 3].

The functional versatility of EVs is largely attributed to their diverse cargo, which includes proteins, lipids, RNA, and DNA [4, 5]. This cargo can reflect the cellular origin and state, enabling EVs to modulate recipient cell behaviour by transferring bioactive molecules [6]. EVs can for example mediate the horizontal transfer of mRNA and microRNA, which can influence gene expression in target cells [7–10]. Additionally, EVs can trigger signalling cascades that affect cellular processes such as proliferation, differentiation, and apoptosis [3, 6].

EVs have a large potential for clinical applications and are being explored for diagnostic, prognostic, and therapeutic purposes. They are attractive biomarker candidates in liquid biopsies due to their change in composition and/or secretion rate depending on the pathophysiological condition. Moreover, the natural biocompatibility and ability of EVs to cross biological barriers, including the blood-brain barrier, position them as promising vehicles for targeted drug delivery [3, 11, 12].

Despite the growing interest in EVs, several challenges remain in the field, including the standardisation of isolation methods, characterization techniques, and understanding their mechanisms of action in vivo. In the past decade the International Society for Extracellular Vesicles has approached these challenges by publishing recommendations and guidance on EV-related studies [13–15]. Nevertheless, despite these efforts, our understanding of EV functions in vivo remains in its early stages. Here progress has been made by utilising the advantages of small animal models such as *Drosophila*, *C.elegans* and the zebrafish to live image and study EVs in vivo [16–21].

Zebrafish have become an increasingly popular model for studying EVs due to their genetic similarities to humans, transparent embryos, and rapid development [16, 17, 19, 22]. Furthermore, as the zebrafish immune system resembles the human immune system more closely compared to invertebrate models, it is an attractive model to study communication via EVs between immune and cancer cells. Of note here, is the transparency of zebrafish embryos and larvae which facilitates real-time in vivo imaging and manipulation of EVs, thereby enhancing our understanding of their role in intercellular communication [18]. However, it’s important now to complement imaging with robust and reproducible methods to isolate, quantify and characterise EVs from larval zebrafish. While previous studies have utilised methods to isolate and characterise EVs from larval zebrafish, these methods do not allow accurate quantification of released EVs as these protocols impact on cell viability, which hinders exclusive isolation and quantification of secreted EVs. This is of crucial importance, as to understand EV function we need to interfere with their release using compounds or genetic approaches and be able to verify the reduction in the amount of released EVs. Hence, we set out to develop a method that allows the isolation of EVs from larval zebrafish without impacting cell viability. Ensuring consistent isolation of released EVs is essential to allow quantification and further analysis. By comparing different methods for tissue digest, followed by an optimised protocol for size exclusion chromatography (SEC) and characterisation of EVs via Nanoparticle Tracking Analysis (NTA) and transmission electron microscopy (TEM), we present a protocol that does not affect cell viability. This protocol not only allows quantification of the amount of released EVs but also results in purified EVs which can be used for downstream applications. Finally, we describe a method to label EVs in a cell type specific manner and show that using our newly developed isolation protocol we can detect these labelled EVs within the total population of purified EVs.

## Materials and methods

### Zebrafish maintenance

Animal studies were approved by the University of Edinburgh Ethical Review Committee and by the Home Office under the Scientific Procedure Act 1986. All zebrafish strains were maintained at the zebrafish facility of the Queen’s Medical Research Institute, University of Edinburgh according to established standard conditions outlined by Westerfield (2000) [23]. All embryos were obtained through natural spawning from adult Et(zic4:GAL4TA4,UAS: mCherry) (Distel, Wullimann and Köster, 2009), Tg(UAS: TagBFP2-HRasV12) (Sieger Lab, University of Edinburgh) and Et(zic4:Gal4TA4,UAS: mCherry; UAS-CD63-eGFP) (Sieger Lab, University of Edinburgh) zebrafish strains, and were kept at 28.5 °C in embryo medium (E3) with a 14-h light/10-h dark cycle. To prevent pigmentation, embryos were treated with 200 µM N-phenylthiourea (PTU) from the end of the first developmental day for the duration of the experiment.

### Cell isolation

To effectively perform cell isolation from 4 days post fertilisation (dpf) zebrafish larvae, three different dissociation strategies were implemented: (a) Collagenase Type I (ThermoFisher, 17018029) treatment [17], (b) mechanical digestion using a homogenizer [24, 25] and (c) enzymatic digestion utilizing *Bacillus Licheniformis* protease (Merck, P5380-100MG) [26].

In the initial step, for all three strategies, anaesthetised 4 dpf zebrafish larvae were carefully placed in 60 mm x 15 mm petri dishes (Corning™, 351007). These petri dishes were filled with ice-cold E3 solution containing 450 µM MS222 (Sigma, A5040). Subsequently, the heads of the larvae were surgically excised using micro-scissors [24, 25]. For the:

- Collagenase Type I treatment: the larval heads were incubated with 200 mg/mL Collagenase I (ThermoFisher, 17018029) dissolved in filtered 1x PBS. The incubation occurred at 29 °C for 45 min, during which the sample was placed on a thermomixer (VorTemp™ 56 Shaking incubator) set to 300 rpm. Trituration was performed every 10 min using a 200 mL filter tip (x20 up/down). Once large cell clumps had dissociated, EDTA (VWR, E177-500ML) was added to stop the enzyme reaction, achieving a final EDTA concentration of 10 mM.
- Mechanical digestion method: the heads were transferred to ice-cold medium A (1x HBSS (Gibco, 14170-088), 15 mM Hepes (Gibco,15630-056), 25 mM Glucose (Sigma, G8644-100ML)) and then manually homogenised on ice using a 1 mL glass homogenizer [25].
- Bacillus Licheniformis protease (Merck, P5380-100MG) enzymatic digestion: truncated heads were incubated with a solution containing 10 mg/mL Bacillus Licheniformis protease (Merck), 5 mM CaCl2, and 125 U/mL DNAse (NEB, M0303S) in filtered 1x PBS at a temperature of 4 °C for 25 min under slow agitation. Every 5 min the sample was triturated with 1000 ml tips, x10 up/down. EDTA (VWR, E177-500ML) at a final concentration of 10 mM was added to stop the enzyme activity after the larger cell clumps had dissociated.

After obtaining homogeneous single cell suspensions using all three strategies, the resulting cell mixture was passed through a 40 μm cell strainer and collected in a 1.5 mL Eppendorf tube. Subsequently, the cells were centrifuged at 300 g for 10 min at 4 °C. The supernatant was used for EV isolation studies and the pellet was resuspended in 22% percoll solution (GE Healthcare,17-0891-02) overlaid by 1x PBS. The mixture was centrifuged at 950 g without break for 30 min at 4 °C. The collected pellet was washed twice with cold medium A, supplemented with 2% normal goat serum (NGS) (Cell Signalling, 5425 S). Each washing step involved centrifugation at 300 g for 10 min at 4 °C. Finally, the isolated cells were resuspended in ice-cold medium A containing 2% NGS and the viability of the cells was evaluated.

### Flow cytometry

The isolated cells were stained with DRAQ7 (ThermoFisher, D15106) at a final concentration of 1 mg/mL. DRAQ7 is a fluorescent DNA dye that selectively binds to the DNA of cells with compromised membranes, characteristic of dead or dying cells. Flow cytometry analysis was performed using the Attune NxT Flow Cytometer. Initially, a segregation based on cellular size (FSC-A) and granularity (SSC-A) was employed to separate cells from debris. Subsequently, single cells were distinguished from doublets or cell clusters by evaluating parameters (FSC Singlet; SSC Singlet). From the population of single cells, a gating strategy was employed to distinguish viable cells (DRAQ7−) from dead cells (DRAQ7+). Flow cytometry data was generated with support from the IRR Flow Cytometry and Cell Sorting Facility, University of Edinburgh. For analysis of the results FlowJo Software 10.8.1 (Treestar, Ashland, OR) was used.

### Separation of EVs from zebrafish larvae by size-exclusion chromatography (SEC)

The obtained supernatants from the cell isolation process were further processed to isolate the EVs. During this stage, four distinct sample preparation methods were employed with different combinations of differential centrifugation and ultrafiltration (UF) (Amicon Ultra-4 10 kDa centrifugal filter (Merck, UFC801008)) (as detailed in Fig. 2A). All strategies were named based on the latest step of EV sample preparation: (a) 2000 g, (b) 10,000 g, (c) 2000 g + UF (10 kDa), (d) 10,000 g + UF (10 kDa). For all strategies, the Izon automatic fraction collector (AFC), utilising principles of size exclusion chromatography (SEC), was used to separate EVs, specifically through a 70 nm generation 2 qEV column (Izon Science, ICO-70).

The isolation of EVs followed the guidelines specified for the SEC procedure. In brief, the SEC column was first thoroughly rinsed with filtered 1x PBS. Following this, a sample with a final volume of 500 µl was applied on top of a qEV column (Izon Science). Subsequently, the column was filled with filtered 1x PBS. The pre-elution buffer volume is specified in the experimental setup section of the results. A total of 13 fractions were then carefully collected, with each fraction having a volume of 400 µl (Izon AFC V2 User Manual). Depending on the specific requirements of the experiments either individual fractions were utilised, or alternatively, multiple fractions were pooled for further study. In addition, pooled fractions were also centrifuged at 4000 g for 20 min at room temperature using an Amicon Ultra-4 10 kDa centrifugal filter to concentrate EVs. Prior to this concentration step, the Amicon Ultra-4 filters were pre-rinsed with 3 ml of filtered 1x PBS and then centrifuged at 4000 g for 15 min. The isolated EV samples were aliquoted and stored at −80 °C.

### Nanoparticle tracking analysis (NTA)

Number of particles and particle size distribution were measured using a ZetaView^®^ TWIN NTA system. The instrument was calibrated using 100 nm polystyrene (PS) beads (Particle Metrix, 110 − 0020) and 100 nm fluorescent Yellow-Green (YG) nanoparticles (Particle Metrix, 120 − 0102). The calibration standards were diluted in filtered cell culture grade water (Corning, 25-055-CV). In contrast, the EV samples were appropriately diluted with a filtered 1x PBS solution to prepare them for analysis. The acquisition parameters employed were as follows: sensitivity: 80, shutter: 100, minimum brightness: 30, minimum size: 10, maximum size: 1000. Videos were captured at 30 frames per second. A sensitivity setting of 95 was used for fluorescence-labelled EVs. Data acquisition and post-acquisition analysis were performed using ZetaView software.

### Protein assay

To quantify the proteins in collected fractions, the “BCA Protein Assay” kit (Thermo Scientific™, 23225) was employed according to the manufacturer’s protocol. In summary, Bovine Serum Albumin (BSA) was prepared in various concentrations (25, 125, 250, 500, 750, 1000, 1500, 2000 µg/mL) and utilized to create a range of protein standards. The BCA working solution was prepared by combining reagent A and B solutions in a 50:1 (v: v) ratio. Subsequently, 200 µL of the working solution was mixed with 25 µL of the EV sample or 25 µL of the standards and then incubated at 37 °C for 30 min. The absorbance of the samples was measured using a microplate reader (SpectraMax i3) at 562 nm wavelength. A standard curve was generated for determining the concentration of protein in each fraction.

### SDS-PAGE and western blot

To perform the western blot, identical volumes (40 µl) of isolated EVs from each fraction were lysed using the 10X RIPA buffer (Merck, 20–188). A volume of 3 parts of extracted EV lysate was diluted in 1 part of 4X Laemmli Sample Buffer (Bio-Rad, 1610747), and beta-mercaptoethanol (final concentration 1 mM) (Life Tech, 31350010) was added. These EV lysates were then heated at 60 °C for 30 min and briefly centrifuged at maximum speed for 5 s to collect condensation. Equal volumes (20 µl) of denatured samples were loaded onto a 4–15% pre-cast polyacrylamide gel (Bio-Rad, 4561085) and resolved by gel electrophoresis at 120 V for 1.5 h using 1X Tris-Glycine SDS (Thermo Scientific™, LC75-4). Proteins were subsequently transferred onto a PVDF membrane (Bio-Rad, 1620262) using a wet tank transfer system (Bio-Rad) with 1X Bolt™ Transfer Buffer (Thermo Scientific™, BT00061). The membrane was dried for 30 min. Subsequently, the membrane was blocked with a solution of 5% non-fat milk (VWR, A0830.0500) in Tris-buffered saline/Tween 20 (0.1%) (TBST) for 1 h prior to an overnight incubation at 4 °C with a rabbit anti-GFP primary antibody (1:2000) (Invitrogen, A11122). The membrane was washed five times with TBST + Milk 5% for 2 min, and incubated with anti-rabbit HRP-secondary antibody (1:2000) (Jackson ImmunoResearch, 111-035-144) for 1 h at RT. Following this, the membrane was washed with TBST + 5% milk, TBST, then TBS five times for 2 min each. Protein detection was accomplished using an enhanced chemiluminescent (ECL) detection kit (Fisher Sci UK, 12644055). The kit solutions were combined in a 1:1 ratio, and the membrane was exposed to this mixture in a light-protected environment for 5 min. The mixture was removed from the membrane, and the membrane was imaged using the Li-Cor (Odyssey) system. The protein ladder (Bio-Rad, 1610374) was visualized using fluorescent detection, while bands of interest were detected using chemiluminescence, following the manufacturer’s guidelines (Odyssey CLx Application Protocols Manual).

### Transmission electron microscopy (TEM)

TEM analysis was conducted using the Joel-TEM 1400 Plus TEM system. The EV sample (≅ 1–2 × 10^10^ particle/mL) was fixed in 2% PFA (Fisher Scientific) at room temperature. A drop of the suspension was deposited on a Formvar/Carbon 200 mesh copper grid (TAAB, FO77/100) for 10 min. Surplus solution was removed with a filter paper touched to the edge of the grid. A drop of 1% aqueous uranyl acetate was allowed to settle for 1 min and then removed by touching the edge of the grid with a filter paper. The grids were then air dried. The samples were then observed in a JEOL JEM-1400 Plus TEM. A GATAN OneView camera was used for representative images. TEM imaging was conducted with support from the Discovery Research Platform for Hidden Cell Biology, University of Edinburgh.

### GW869 treatment to block EV formation and release

To prevent EV formation and release, GW4869 (Generon, A11974-50), dissolved in DMSO, was used. Zebrafish larvae at 2 dpf were first treated with 1 mg/mL pronase (in E3 medium) (Roche, 10165921001) for 10 min under gentle agitation to remove the chorion. The larvae were then washed three times with E3 medium. Dechorionated larvae were subsequently treated for 48 h with 1% DMSO/10 µM GW4869, 1% DMSO/20 µM GW4869 or 1% DMSO as a control. For initial toxicity trials larval fish were monitored for 48 h focusing on developmental abnormalities, including pericardial oedema, yolk sac oedema, blood accumulation, tail abnormalities and necrosis. These trials revealed no developmental abnormalities or mortalities, confirming that larval zebrafish tolerated both concentrations (10 µM and 20 µM) of GW4869 treatment well. Following the 48 h treatment, EVs were extracted using our new protocol. Fractions F2 to F5 were pooled for analysis.

Tissue weight was used to normalise the EV amount. For tissue weight calculation, empty 1.5 mL Eppendorf tubes were initially weighed using a 4-digit analytical balance. After head truncation, the heads were transferred to these 1.5 mL Eppendorf tubes. The transfer liquid was removed with a syringe, and the tubes were weighed again using the 4-digit analytical balance. Tissue weight was determined by subtracting the blank weight from the measured value.

### Plasmid DNA injection

To visualize EVs we used a fusion construct of zebrafish CD63 and GFP. To induce expression of CD63-GFP, Et(zic4:GAL4TA4,UAS: mCherry) zebrafish embryos or Et(zic4:GAL4TA4,UAS: mCherry)/Tg(UAS: TagBFP2-HRASv12) embryos were injected with pDEST-UAS-CD63-GFP-pA at the one cell stage using the PV820 Pneumatic PicoPump from World Precision Instruments (WPI, USA). Injection solution containing 60 ng/µL plasmid DNA, 20 ng/µL Tol2 capped mRNA, 1 M KCl and to facilitate visualization of the injection 0.1% phenol red (Sigma, Sigma-Aldrich, P5530) was injected. Larvae were screened for positive transgene expression at 2–3 dpf. Only embryos exhibiting a normal development were selected for the required experiments.

### Live fluorescent imaging

For imaging, 4 dpf zebrafish larvae were anaesthetised in 450 µM MS222 (Sigma, A5040) and carefully positioned in a dorsal-up orientation and embedded in 1.5% LMP agarose (Invitrogen, 16520) in E3 medium on a 1.5 mm-thick glass coverslip (SLS, MIC3114). A microscope glass slide was bordered with a high vacuum grease (Sigma, Z273554-1EA) and then filled with E3 medium containing 275 µM MS222 (Sigma, A5040). All single-time points (with z-step = 2 µm) and time-lapse Z-stacks (with z-step = 0.2 µm and Δt = 11’’) were acquired with the LSM880 confocal laser scanning microscope, and processed using the default Airyscan processing settings (Zen Black 2.3, Zeiss, Germany). Confocal images were captured using a 20x objective lens (Plan Apochromat 20x air, NA = 0.8). Super-resolution imaging was obtained using the Zeiss LSM880 microscope with Airyscan in super-resolution mode using a 63x objective lens (either Zeiss C-Apochromat 63x water-dipping, NA = 1.2 or Plan Apochromat 63x oil-dipping, NA = 1.4). EV released by radial glial progenitor cells were imaged along the TPZ at the level of the optic tectum. Laser lines at 405 nm, 488 nm, 561 nm and 633 nm were utilised to illuminate the samples. Live fluorescent imaging was carried out with support from the UK Zebrafish Imaging and Screening Facility, University of Edinburgh.

### Image analysis

The images obtained through confocal microscopy or TEM were analysed using the Imaris software 10.1.1 (Bitplane, Switzerland) and ImageJ/FiJi 2.14.

### Statistical analysis

Statistical analysis and graph generation were conducted using GraphPad Prism Software version 9.5.1. Data was tested for normality using the Shapiro-Wilk test. Unpaired two-tailed Student’s t-tests were employed for the comparison of two experimental groups. In cases of multiple comparisons, a comprehensive statistical assessment was performed using a one-way ANOVA test or two-way ANOVA test, followed by the application of Tukey’s or Šídák’s multiple comparisons test. P-value < 0.05 was used as the criterion for statistical significance.

## Results

### *Bacillus Licheniformis* protease tissue digestion results in optimal cell viability

**Fig. 1:**
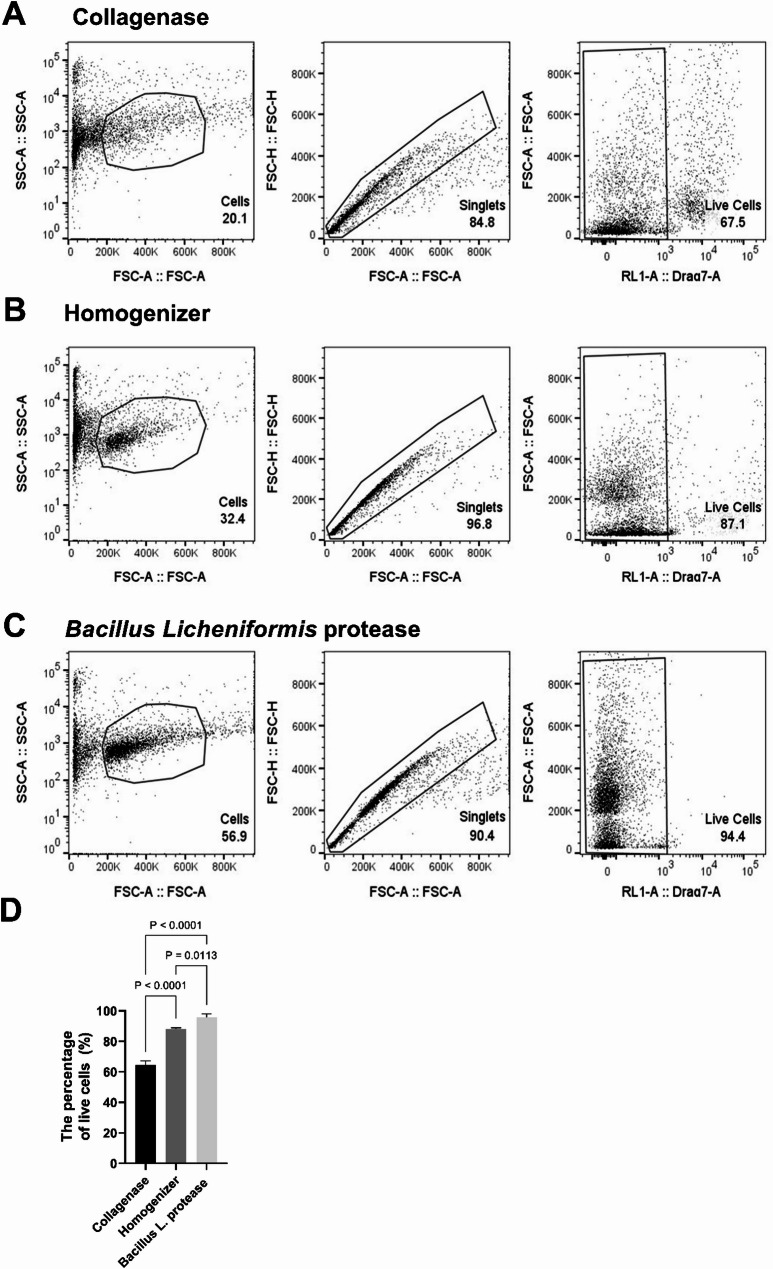
The Bacillus licheniformis protease enzyme is the most efficient tissue dissociation method for preserving cell viability. Cells were isolated from 4 dpf larval heads (*n* = 50 and *N* = 2). Images show representative flow cytometry gating strategy designed to identify all types of brain cells and singlets through the utilisation of forward scatter (FSC) and side scatter (SSC). Non-viable cells were excluded by DRAQ7 labelling after treatment with different methods: (**A**)Collagenase, (**B**) Homogenizer, and (**C**) Bacillus Licheniformis protease, (**D**) The overall percentage of live cells following the distinct techniques. Statistical analyses revealed significant differences between the Bacillus Licheniformis protease enzyme and both the homogenizer (*P* = 0.0113) and collagenase treatment (*P* < 0.0001). One-way ANOVA statistical analysis and Tukey’s multiple comparisons test were used to calculate P values. P-values were indicated if they are statistically significant (*P* < 0.05). Error bars represented the standard error

Our aim was to develop a robust and replicable approach for extracting EVs from larval zebrafish tissues without affecting cell viability. To achieve this aim, we focused on larval heads as these present one of the most challenging tissues due to complex tissue composition including the presence of myelinated axons. Based on our previously developed protocol for cell isolation from larval heads, 50 larval heads were excised using micro-scissors and subjected to several tissue dissociation techniques [24, 25]. For tissue digestion, it was crucial to identify a method that does not affect cell viability. This is important, since subsequent analyses on concentration and content of released EVs would be impacted if cell viability was impaired and cellular membranes were ruptured. Previous studies have used the collagenase enzyme to digest the tissue prior to the separation of EVs [17, 19, 27, 28]. Therefore, we tested the collagenase enzyme in comparison to two other methods: mechanical digestion using a homogenizer [24, 25] and *Bacillus Licheniformis* protease treatment [26]. After obtaining homogeneous cell suspensions using all three strategies, the resulting cell mixture was passed through a 40 μm cell strainer and centrifuged at 300 g for 10 min at 4 °C. After this step resulting supernatants were subjected to EV purification. However, to assess cell viability, the cell pellets were resuspended in 22% percoll solution overlaid by 1x PBS and cells were separated as described previously (see methods; [24, 25]). Cell viability was then assessed with DraQ7 staining followed by flow cytometry (Fig. 1).

The comparison of the three methods revealed that collagenase had a substantial impact on cell viability, resulting in a percentage of live cells at 64.4% ± 1.6% (Fig. 1A, D). Conversely, when employing a homogenizer, a modest effect on cell viability was observed, yielding a percentage of viable cells at 88% ± 0.5% (Fig. 1B, D). Notably, the viability rate increased to 95.7% ± 1.3% with *Bacillus Licheniformis* protease treatment (Fig. 1C). In conclusion, the *Bacillus Licheniformis* protease enzyme provided the most favourable outcome, exhibiting a minimal effect on cell viability in comparison to the homogenizer and collagenase treatment (Fig. 1D). Based on these results, the *Bacillus Licheniformis* protease enzyme was considered the most suitable method for tissue dissociation during the extraction of EVs.

### Separation and characterization of EVs

**Fig. 2:**
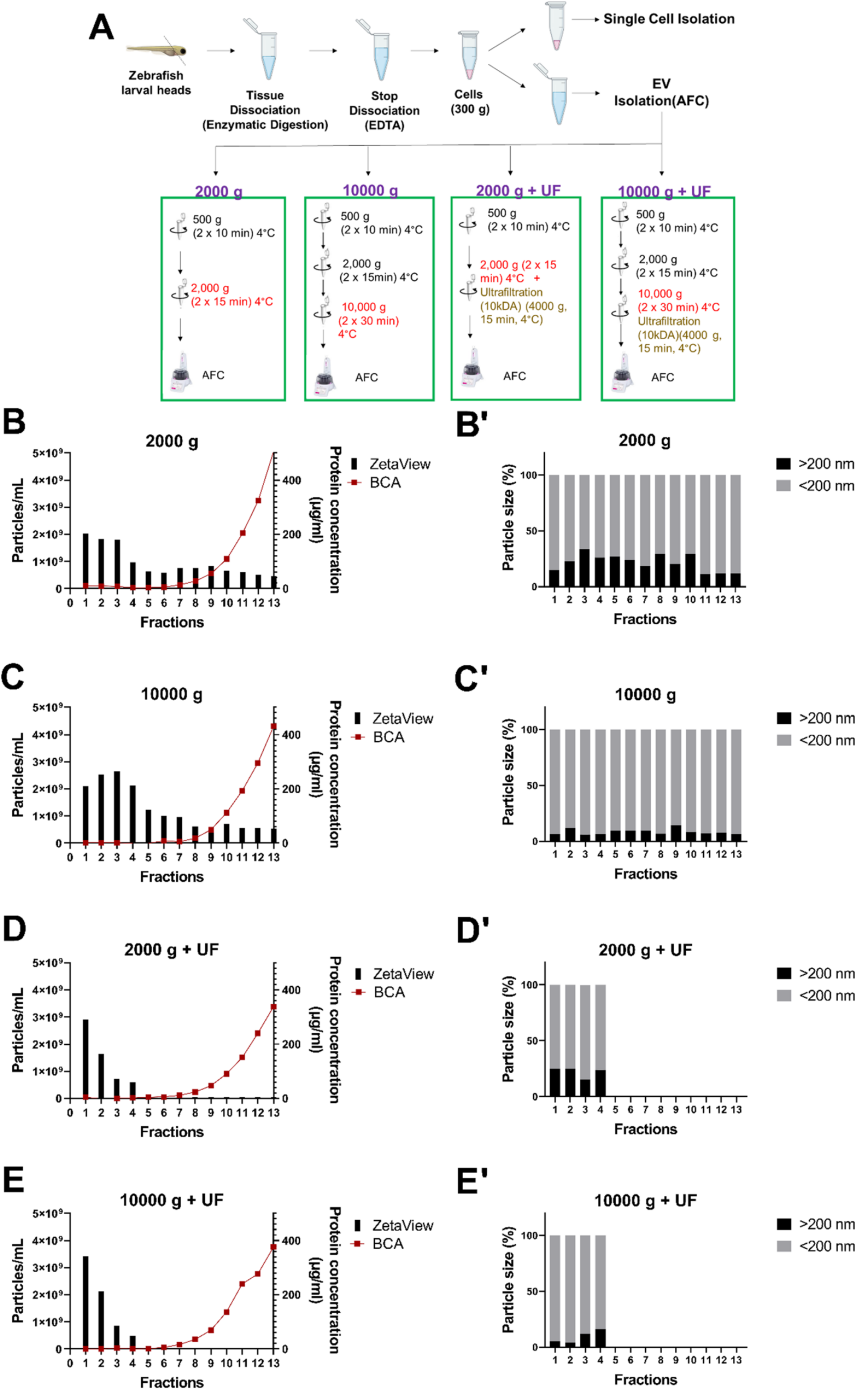
The combination of centrifugation and UF is the most appropriate approach for the extraction of EVs from larval zebrafish heads. **A** Strategies tested to isolate EV samples from zebrafish larval heads: Following head truncation, tissue dissociation was accomplished using the Bacillus Licheniformis protease enzyme, and the resulting single cell solutions were centrifuged. The obtained cell pellet was used for single cell isolation. For EV isolation, the supernatant was further processed by four different strategies which are combinations of differential centrifugation and ultrafiltration. Each approach is named according to the latest stage of preparing the EV sample. For instance, 2000 g represents the final step before the SEC protocol was conducted. The same SEC protocol was applied to allstrategies tested. **B-E** Quantification of particle amounts via NTA and quantification of protein levels via BCA assay in each fraction for the different sample preparation strategies. EVs were extracted from 4 dpf larval heads (n = 50 and N = 1). **B **2000 g **C** 10000 g. NTA analysis of both 2000 g and 10000 g sample preparations revealed the presence of particles in all fractions. Notably, a progressive reduction in particle quantity was observed towards the later fractions. Also, these groups exhibited EV-rich and protein-low characteristics in the initial fractions [1–6], while the later fractions [7–12] displayed low EV content but high protein concentration. **D** 2000 g + UF, **E** 10000 g + UF. In these groups, particles were detected in the first fractions (F1-F4) only. BCA results indicated that these early fractions had low protein concentrations. **B'-E'** Analysis of particle size via NTA under different sample preparation strategies. Particles smaller than 200 nm were generally detected in the EV-containing fractions from all sample preparation strategies. Particles larger than 200 nm were also detected. The percentage of particles larger than 200 nm was **B'** 2000 g (21.6% ± 2.1%), **C'** 10000 g (8.5 ± 0.7%), **D'** 2000 g + UF (22.2% ± 2.3%), **E'** 10000 g + UF (9.4 ± 2.9%). These values represented the average of all the fractions in which particles were present

A significant challenge in isolating EVs is achieving this without compromising their biological functionality. Although ultracentrifugation (UC) and density gradient ultracentrifugation (DGUC) are widely employed for EV isolation, these techniques may negatively impact EV functionality due to the high centrifugal forces applied to the samples and the use of various density gradient compounds [29, 30]. In contrast, previous studies have demonstrated that SEC preserves the biophysical and functional properties of EVs [31–33]. Hence, we decided to use SEC to separate EVs from digested zebrafish larval head tissue. This approach has also been shown to be effective in separating EVs from smaller non-vesicular structures from biological fluids such as protein aggregates which can interfere with downstream analyses like proteomic and lipidomic studies [34, 35]. Using SEC, it’s important to note that the fractions containing EVs may vary depending on the sample type or preparation. In that case, it becomes vital to investigate both the non-vesicular component and EV content of each fraction. Hence, the aim of this step was to identify a specific sample preparation strategy to ensure the extraction of high quality EVs from zebrafish larval heads. We dissected and digested larval heads as described above using the *Bacillus Licheniformis* protease enzyme, passed the resulting cell mixture through a 40 µm cell strainer and centrifuged at 300 g for 10 min at 4 °C. The supernatants obtained after the cellular dissociation were then further processed. We tested four different sample preparation strategies. These were combinations of differential centrifugation and ultrafiltration (UF) (Fig. 2A: 2000 g, 10000 g, 2000 g + UF (10 kDa), 10000 g + UF (10 kDa). The same SEC protocol was then applied to all strategies using a 70 nm generation 2 qEV column (Izon Science) on an automated fraction collector (Izon Science). As at this stage it was not clear which sample preparation strategy was successful, we followed recommended settings for EVs from serum samples (manufacturer guidelines, Izon Science). Here, the elution buffer volume prior to collection of EVs was set at 2.9 mL and 13 fractions of 400 µL each were collected and analysed using the NTA to determine particle concentrations and sizes. Simultaneously, the presence of protein contamination was assessed utilising the BCA assay method (Fig. 2B, C,D, E).

Based on EV separation strategies for serum samples, the early fractions [1–4] were expected to provide EV-rich, protein low content, while the following fractions [5–13] were expected to be characterised by a progressive increase in the amount of protein contaminant but low EV presence. Contrary to these expectations, our NTA results obtained from the 2000 g and 10,000 g sample preparations revealed the presence of particles across all fractions (Fig. 2B, C). In both sample preparation approaches, the early fractions (F1-F4) exhibited an approximate particle concentration of 2 × 10^9^ particles/mL. A gradual decrease in particle concentration was observed towards the late fractions, with the final fractions (F10-F13) reaching a concentration of 5 × 10^8^ particles/mL (Fig. 2B, C). Remarkably, the 2000 g + UF (10 kDa) and 10,000 g + UF (10 kDa) samples exhibited a trend, where particles were detected in the first fractions only (F1-F4) (Fig. 2D, E). In both experimental groups, the particle count in the initial fraction (F1) was approximately 3 × 10^9^, gradually decreasing to 5 × 10^8^ by the fourth fraction (Fig. 2D, E). As expected, the protein analysis results conducted by BCA assay showed that the initial fractions [1–6] exhibited low protein concentration, with 0–10 µg/mL in all sample preparation strategies (Fig. 2B-E). In contrast, the later fractions [7–13] displayed high protein concentrations, reaching approximately 400 µg/mL for the thirteenth fraction in all test groups (Fig. 2B-E). In summary, based on particle concentration and protein concentration, we observed that 2000 g + UF and 10,000 g + UF result in EV-rich and protein low content in early fractions (F1-F4).

In the subsequent analysis, the size distribution of EVs in each fraction was examined based on the NTA results. Here we calculated the percentage of particles > 200 nm as these particles may be large EVs, defined as > 200 nm according to the latest guidelines established by the International Society for Extracellular Vesicles [14, 15], or they could be EV agglomerates or small cell debris. The EV-containing fractions from all sample preparation strategies exhibited the presence of particles smaller than 200 nm. Notably, particles larger than 200 nm were also detected (Fig. 2B’, C’, D’, E’). Specifically, the test groups subjected to 2000 g and 2000 g + UF showed a higher percentage of particles exceeding 200 nm, with an average of 21.6% ± 2.1% and 22.2% ± 2.3% across all fractions, respectively (Fig. 2B’, D’). In contrast, the test groups subjected to 10,000 g and 10,000 g + UF demonstrated a lower percentage of particles exceeding 200 nm, with values of 8.5% ± 0.7% and 9.4% ± 2.9%, respectively (Fig. 2C’, E’).

In summary, while the 2000 g + UF and 10,000 g + UF sample preparation strategies showed a similar trend in generating EV-rich and protein-low content in early fractions (F1–F4) based on NTA and BCA measurements, differences were detected in the percentage of particles > 200 nm. However, given the known limitations of these methods such as BCA’s low sensitivity to small or fragmented protein aggregates (⩾20 ug/ml) and NTA’s inability to distinguish between EVs and similarly sized contaminants more detailed analyses were conducted using TEM.

**Fig. 3:**
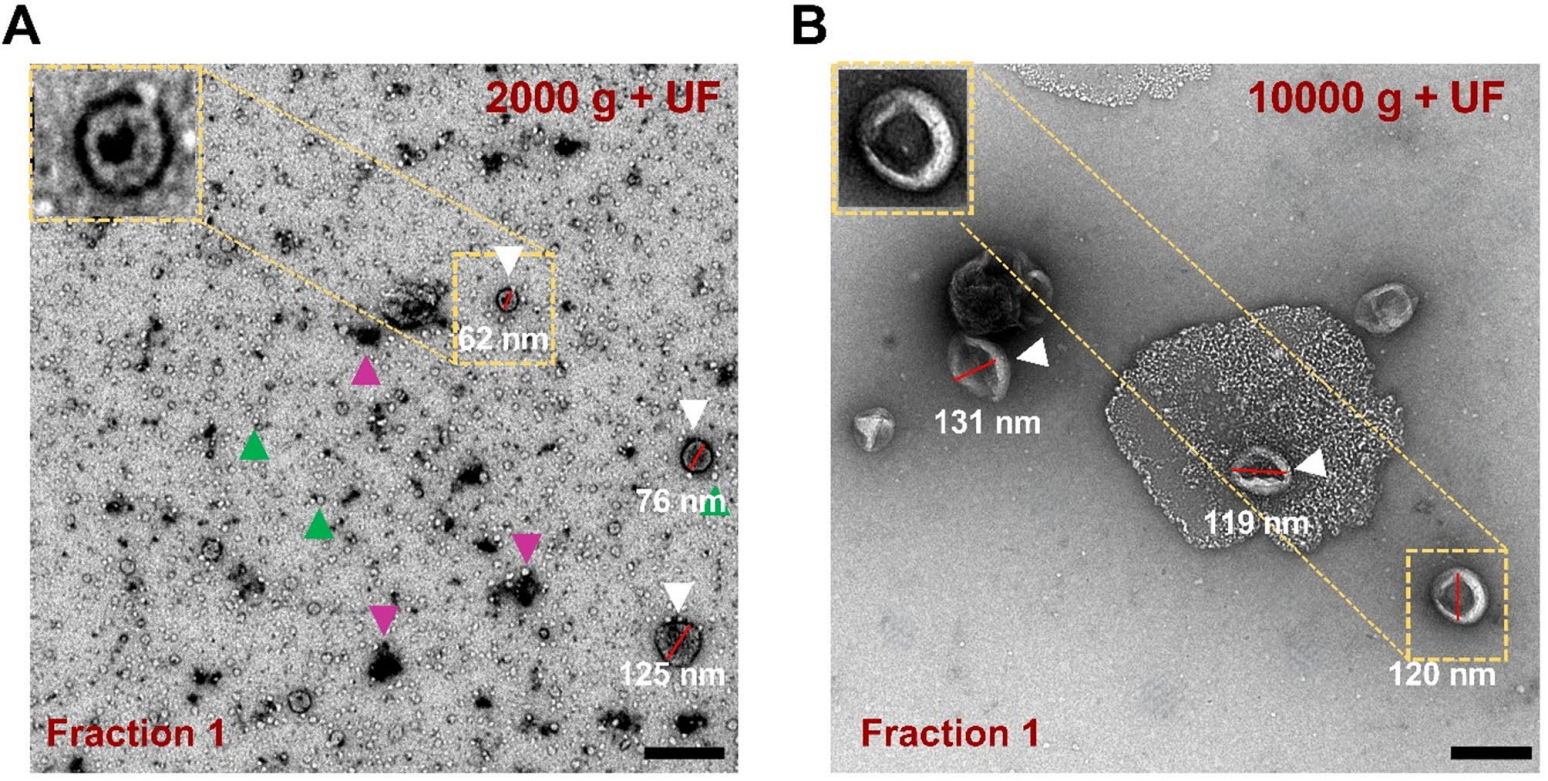
TEM reveals low contamination for the 10,000 g + UF protocol for the extraction of EVs from larval zebrafish heads. TEM analysis (representative images shown) revealed the presence of EVs and these identified EVs were less than 200 nm in size. **A** 2000 g + UF, (**B**) 10,000 g + UF. In particular, the presence of protein aggregates and lipoparticles was observed in the 2000 g + UF test group. The EVs in the 2000 g + UF group appear to display a distinct dark halo that is not observed in the 10,000 g + UF condition, which may reflect a protein corona. EVs (white arrowheads), protein aggregates (magenta arrowheads) and lipoparticles (green arrowheads). Scale bar: 200 nm

The TEM results in both test groups indicated the presence of EVs with a size of less than 200 nm (Fig. 3). However, F1 from the 2000 g + UF preparation contains a considerable amount of protein aggregates and lipoparticles, which are not fully captured by BCA quantification due to its limited sensitivity to small or fragmented protein aggregates (⩾20 ug/ml) (Fig. 3A). Furthermore, the high abundance of lipoparticles may significantly impact the accuracy of NTA measurements by artificially inflating particle counts and affecting size distribution profiles. These findings highlight that despite similar protein trends, particle composition and purity vary between the two preparation methods and should be carefully considered when interpreting downstream analysis. Considering the comprehensive comparisons conducted here and aiming at the isolation of small EVs with minimal contamination (Fig. 3B), we concluded that the 10,000 g + UF test group represents the most appropriate method for the extraction of particles from larval zebrafish heads, which we can define as EVs. This conclusion is based on detection of EVs in fractions 1–4, lower occurrence of particles larger than 200 nm, and a diminished presence of contamination.

**Fig. 4:**
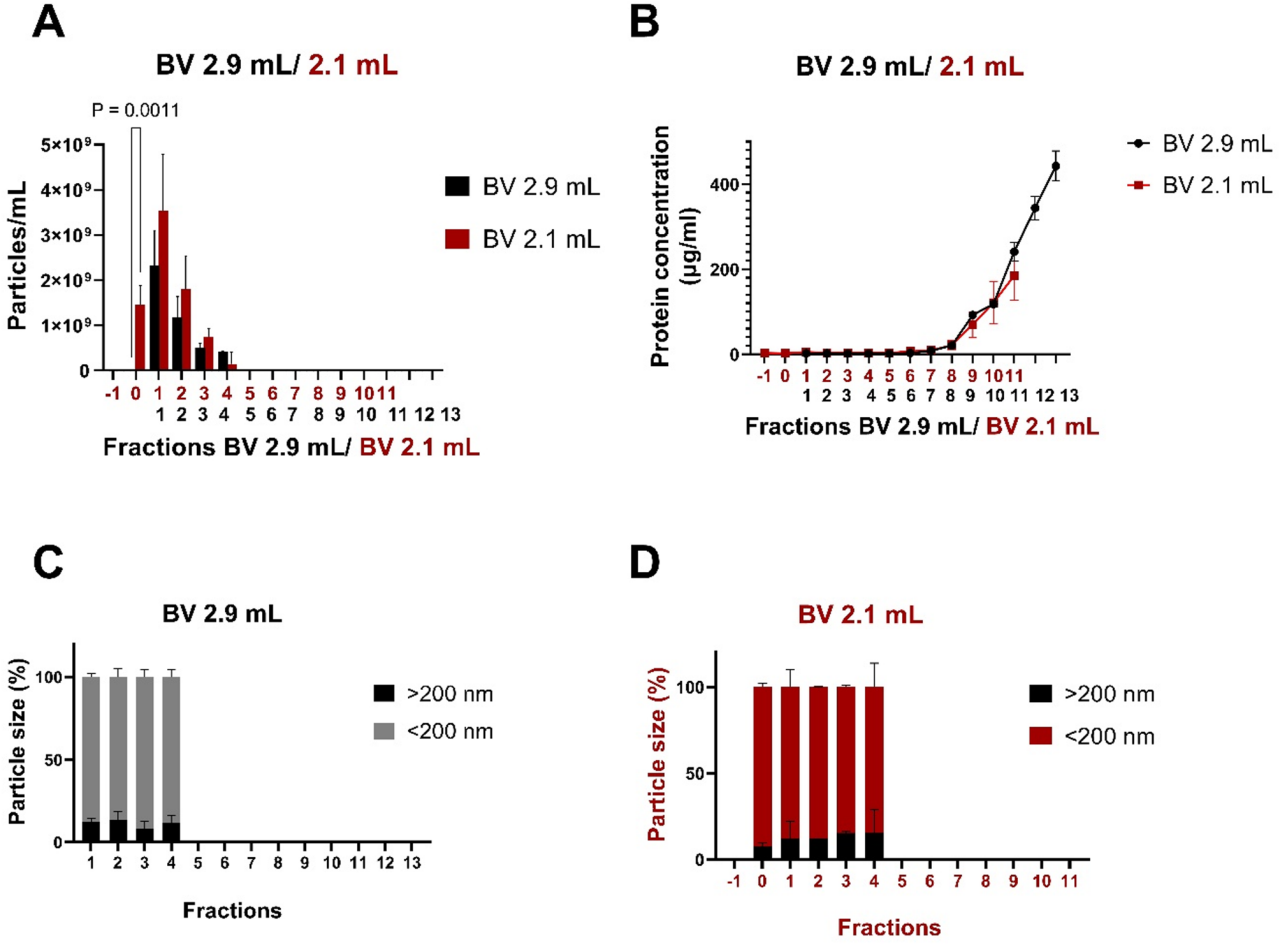
A buffer volume of 2.1 mL represents the most optimal adjustment for EV extraction from larval zebrafish heads. EVs were extracted from 4 dpf larval heads (*n* = 50 and *N* = 2). **A** Quantification of EVs with different buffer volumes. Significant EV amounts (approximately 1.5 × 10^9^ particles/ml) (*P* = 0.0011) were detected in the F2 fraction, which was discarded with the higher buffer volumes (2.9 ml). There was no significant difference in the amount of EVs within the other EV-containing fractions. Two-way ANOVA statistical analysis and Šídák’s multiple comparisons test were used to calculate P values. P-values were indicated if they are statistically significant (*P* < 0.05). Error bars represented the standard error. **B** Protein level analysis of EVs with different buffer volumes. No contamination was detected in the EV-containing fractions in either experimental condition. **C-D** Detection of EV size with different buffer volumes. In both groups, particles smaller than 200 nm were generally detected in the EV-containing fractions. Particles exceeding 200 nm in size were also observed. The proportion of particles larger than 200 nm was **(C)** BV 2.9 ml (11.4% ± 1,1%) **(D)** BV 2.1 mL (10.4% ± 2.4%). These values represent the average values for all the fractions in which EVs were present. Unpaired t-test analysis was done with these values and no significant differences were found in terms of size in both groups

As a final optimization, we decided to test the impact of different buffer volumes before elution during SEC and the impact of the number of larval zebrafish heads used for isolation. For the initial development of the sample collection strategy, we followed established protocols for the purification of EVs from serum samples. Here, the elution buffer volume prior to collection of EVs was set at 2.9 mL, and 13 fractions of 400 µl each were collected. As we have now established the optimal sample preparation strategy, we reasoned that conditions might be different when processing head tissue samples compared to serum samples and decided to reduce the elution volume to test if a certain amount of EVs might be lost with an elution buffer volume of 2.9 ml. Hence, we decided to collect 2 additional fractions (400 µl each), which reduced the elution buffer volume to 2.1 ml and the subsequent 13 fractions were collected and compared to the fractions obtained with a 2.9 ml buffer volume. This modification allowed us to recover two more fractions (F1 and F2), which were eliminated when using the higher buffer volume (2.9 mL). Based on NTA analysis, we did not detect EVs in the F1 fraction when the buffer volume was set at 2.1 ml. In contrast, a substantial amount of EVs (approximately 1.5 × 10^9^ particles/mL) was detected in the F2 fraction which was discarded at higher buffer volume (2.9 mL) (Fig. 4A). There was no significant difference in the amount of EVs observed when comparing other EV-containing fractions (Fig. 4A). Moreover, purification analysis based on the BCA assay revealed no contamination in the fractions containing EVs in both experimental groups (Fig. 4B). As expected, protein contamination was detected towards the last fractions of both experimental groups. Finally, size analysis determined by NTA, revealed that in both groups, the particles in the EV-containing fractions were generally found to be small EVs (< 200 nm) and no significant difference was detected in the average percentage of large particles (Fig. 4C, D). These findings emphasise the sensitivity of the isolation process to the starting material and result in respective buffer volume adjustments. Hence, we decided to utilise a buffer volume of 2.1mL, which results in a fraction F1 free of EVs and fractions F2 to F6 containing EVs.

**Fig. 5:**
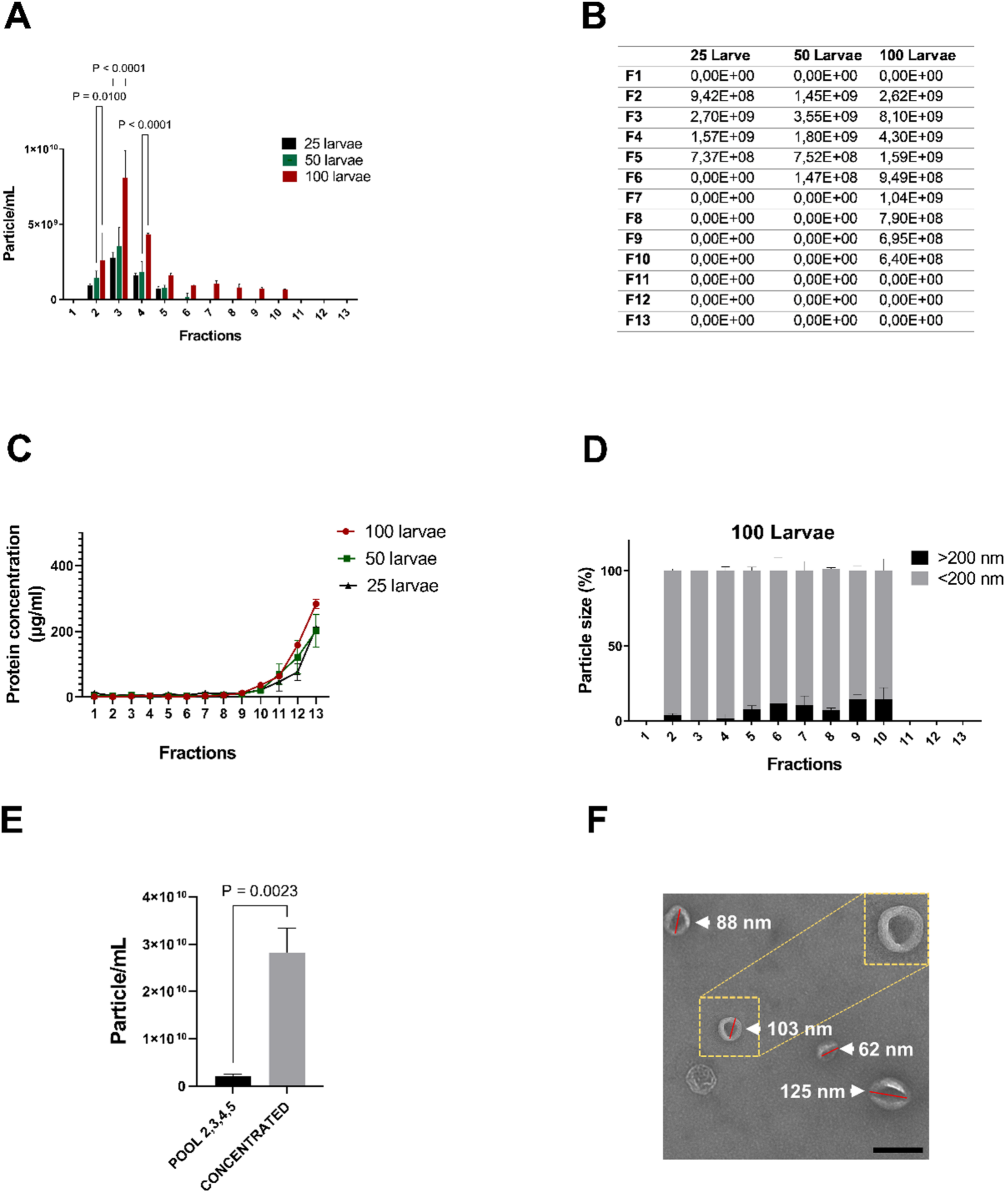
**A**-**D** Utilizing different numbers of zebrafish larval heads impacts on EV quantification. EVs were isolated from 4 dpf larval heads (*n* = 25, *n* = 50, *n* = 100 and *N* = 2) **A-B** The amount of EVs in each fraction from different larval zebrafish heads. No significant change was observed between 25 and 50 larvae. Considerable increase was observed in fraction 2,3 and 4 when 100 larvae were used compared to 50 larvae (*P* = 0.01, *P* < 0.0001, and *P* < 0.0001, respectively) and compared to 25 larvae (*P* = 0.002, *P* < 0.0001, and *P* < 0.0001, respectively (p values not displayed on the graph)) (**C)** Protein contamination in fractions from 25, 50 and 100 larvae. No contamination was found in the initial fractions (F2-F6) in all experimental groups. A significant increase in contamination levels in the last fraction was observed for the 100 larvae experimental set-up compared to 25 and 50 larvae (*P* < 0.0001 and *P* = 0.0003, respectively (p values not displayed on the graph)). **D** Detection of EV size from 100 larval heads. Particles < 200 nm were commonly identified in the EV-containing fractions. Particles > 200 nm were also present. In particular, the proportion of these particles was slightly higher in the last fractions (F7-F11) than in the first fractions (F2-F5). Two-way ANOVA statistical analysis and Tukey’s multiple comparisons test were used to calculate P values. P-values were indicated if they are statistically significant (*P* < 0.05). Error bars represented the standard error. **E-F **Ultrafiltration efficiently concentrates pooled EV fractions. EVs were extracted from 4 dpf larval heads (*n* = 50 and *N* = 2) (**E**) Evaluation of the quantity of the concentrated fractions using NTA. Fractions 2, 3, 4, and 5 were combined to create a pooled sample, which was then concentrated and enriched approximately 10-fold (*P* = 0.0023) through ultrafiltration. Unpaired t-test was used to calculate P values. **F** Demonstration of the purity of concentrated EVs via TEM (representative image shown). Detected EVs were usually < 200 nm. EVs (white arrowheads). Scale bar 200 nm

Furthermore, we tested the potential impact of varying the number of larval zebrafish used. We aimed to understand how the number of larval zebrafish heads might influence the distribution of EVs into different fractions, the total yield and the protein level of isolated samples. Hence, EV extraction was performed utilising 25, 50, and 100 larval zebrafish heads. Based on NTA results, the analysis of 25 larval zebrafish heads revealed the presence of EVs within the F2-F5 fractions (Fig. 5A). In contrast, the examination of 50 larval heads demonstrated the presence of EVs within the F2-F6 fractions. Although no significant difference was observed in the quantity of EVs between these two groups, the use of 50 larvae resulted in slightly higher amounts of EVs (Fig. 5A, B). Conversely, the use of 100 larval heads had a notable impact on the distribution and concentration of EVs in the fractions (Fig. 5A, B). This led to a substantial increase in EV amount, particularly in fractions F2, F3, and F4 compared to the use of 25 larvae (*P* = 0.002, *P* < 0.0001 and *P* < 0.0001, respectively) and 50 larvae (*P* = 0.01, *P* < 0.0001 and *P* < 0.0001, respectively). In addition, it resulted in detection of particles in the later fractions (F7-F11) (Fig. 5A, B). In further analyses using 100 larval zebrafish heads, no protein contaminants were detected in the initial fractions (F1-F6) similar to using 25 or 50 larval heads (Fig. 5C). However, there was a significant increase in the amount of contaminant towards the last fraction, which reached roughly 300 µg/mL compared to approximately 200 µg/mL for samples derived from 25 to 50 larvae (*P* = 0.0003 and *P* < 0.0001, respectively). Size analyses of EVs isolated from 100 zebrafish larval heads revealed the presence of particles larger than 200 nm in almost every fraction which contained EVs. Notably, in the last fractions (F7-F11) the rate of these particles was observed to be slightly higher than in the first fractions (F2-F5) (Fig. 5D).

These results showed that a change in the number of larval heads can affect the distribution, protein level and amount of EVs in the fractions. While 25 larval heads can be used for the isolation process, the total yield from 25 heads is lower compared to 50 and 100 larval heads. Employing 100 larval heads led to the highest yield of EVs, however, this setting also resulted in the appearance of particles in later fractions (F7-F11), which in part showed protein contaminations (F9-F11). Hence, depending on the experimental objectives, the utilisation of different numbers of larvae can be considered; nevertheless, it is recommended to focus on fractions F2-F6 for optimal results. For the isolation of EVs from larval zebrafish heads at 4 dpf our recommendation is using 50 larval heads, which results in a high yield of EVs and optimal reproducibility.

Finally, as for many downstream approaches such as transcriptomics and proteomics, purified EVs need to be concentrated, we pooled EV containing fractions, concentrated them and tested their purity again via TEM. The F2-F5 fractions were pooled and concentrated by the ultrafiltration method. NTA results revealed that the pooled fractions were concentrated approximately 10-fold (*P* = 0.0023) (Fig. 5E). Importantly, this concentration strategy did not impact on the purity of EVs as shown by TEM analysis (Fig. 5F compared to Fig. 3B).

In conclusion, our final protocol consists of the following steps: [1] excision of larval heads [2] tissue digest via *Bacillus Licheniformis* protease treatment (with EDTA addition to stop enzyme activity) [3] passing the cell mixture through a 40 μm cell strainer [4] a series of differential centrifugation steps at 4 °C: 300 g for 10 min; 500 g for 2 × 10 min; 2000 g for 2 × 15 min; 10,000 g for 2 × 30 min [5] Ultrafiltration (10 kDA) 4000 g for 15 min at 4 °C [6] size exclusion chromatography (SEC) via an automatic fraction collector (Izon Science) using a 70 nm generation 2 qEV column (Izon Science, ICO-70), initial elution buffer set to 2.1 mL followed by collection of 13 fractions of 400 µl. Optionally, EV containing fractions can then be pooled and concentrated by centrifugation at 4000 g for 20 min at room temperature using an Amicon Ultra-4 10 kDa centrifugal filter.

### GW4869 inhibits EV release in the larval zebrafish brain

**Fig. 6:**
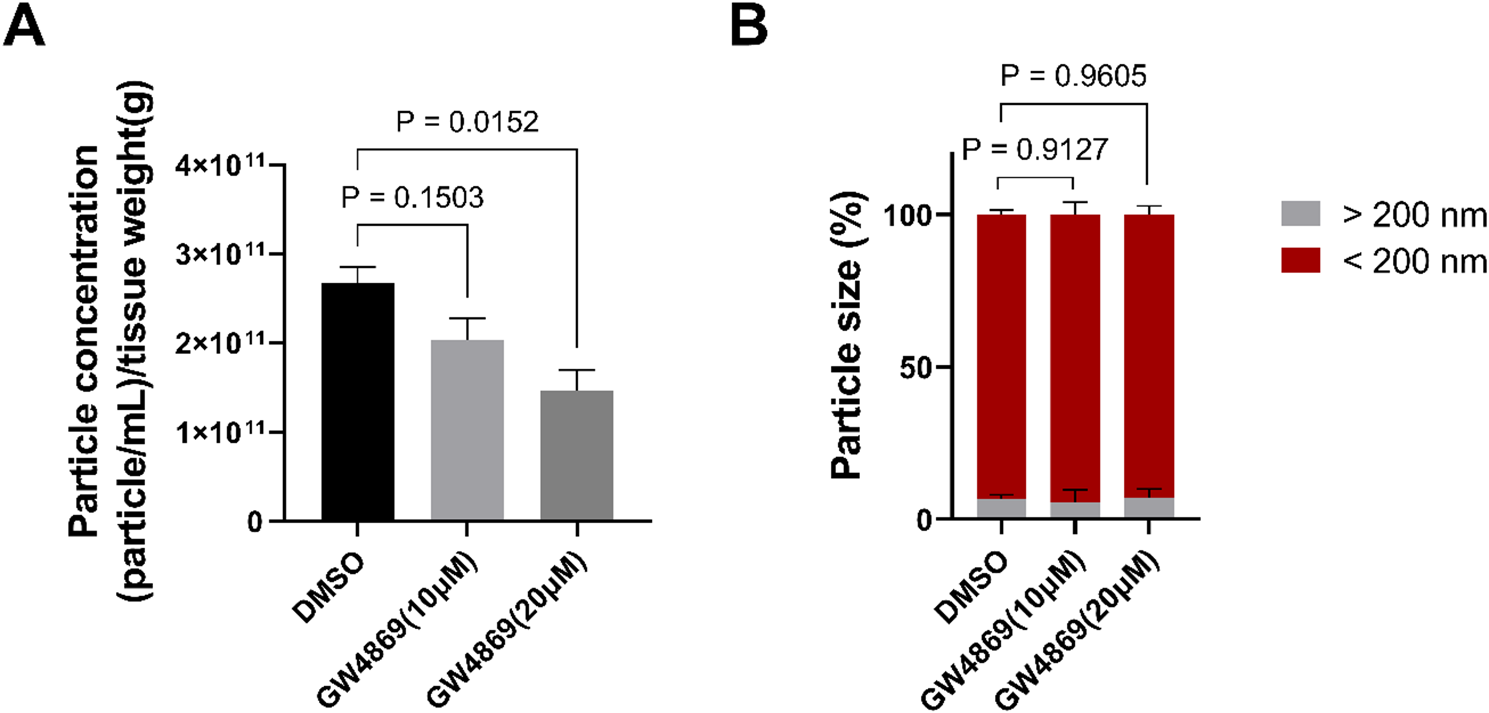
GW4869 treatment inhibits EV release in larval zebrafish heads. After 48 h treatment with GW4869/DMSO or DMSO EVs were extracted from 4 dpf larval heads of zebrafish (*n* = 50 and *N* = 3). Concentration and size analysis of the EVs were evaluated via NTA. The amount of EVs were normalised based on tissue weight. **A** The amount of EVs from DMSO and GW4869-treated zebrafish larvae. Considerable inhibition of the EV number was observed after GW4869 (20 µM) treatment compared to the DMSO-treated group (*P* = 0.0152). **B** Size distribution of EVs from DMSO and GW4869-treated zebrafish larvae. No significant changes in particle size distribution were detected. One-way ANOVA statistical analysis and Dunnetts’s multiple comparisons test were used to calculate P values. Error bars represented the standard error

As we have established a robust and reproducible approach for extracting EVs, we wanted to test if this method is sensitive enough to detect differences upon interference with EV production and release. To address this question, we inhibited EV generation by treating larval zebrafish with GW4869, which inhibits ESCRT-independent biogenesis of EVs [36, 37]. Toxicity trials revealed that larval zebrafish tolerate 20 µM GW4869 treatment for 48 h without showing any adverse effects. Hence, 2 dpf larval zebrafish were either treated with 1% DMSO (control), 1% DMSO/10 µM GW4869 or 1% DMSO/20 µM GW4869 for a duration of 48 h. Each group consisted of 50 embryos and after 48 h of treatment (4 dpf), EVs were isolated using our protocol. Fractions F2 to F5 were pooled for analysis, and concentration and size analysis of the EVs were conducted using the NTA system. Tissue weight was used to normalise the EV amount (see methods). Analysis showed that the 10 µM GW4869 treatment resulted in a 23.7% ± 9.8% reduction in EVs compared to the control group, although this reduction was not statistically significant (Fig. 6A). In contrast, 20 µM GW4869 treatment resulted in a notable inhibition of EVs (*P* = 0.0152) compared to the control group, reducing their levels by almost 45.7% ± 7.8% (Fig. 6A). Further analysis of this drug treatment involved examining the size distribution of EVs based on NTA results. All test groups exhibited particles < 200 nm, and particles > 200 nm were also detected. However, no significant changes in particle size distribution were observed across all drug treatments compared to the control (Fig. 6B).

In summary, these results indicate that GW4869 effectively reduces EV generation in a reproducible and dose dependent manner in larval zebrafish and that our newly developed protocol has the sensitivity to detect a reduction in EVs. Hence, this protocol offers promising potential for future applications, providing a valuable platform for testing new drugs targeting EV-related pathways *in vivo.*

### Cell specific labelling and in vivo imaging of EVs in the larval zebrafish brain

**Fig. 7:**
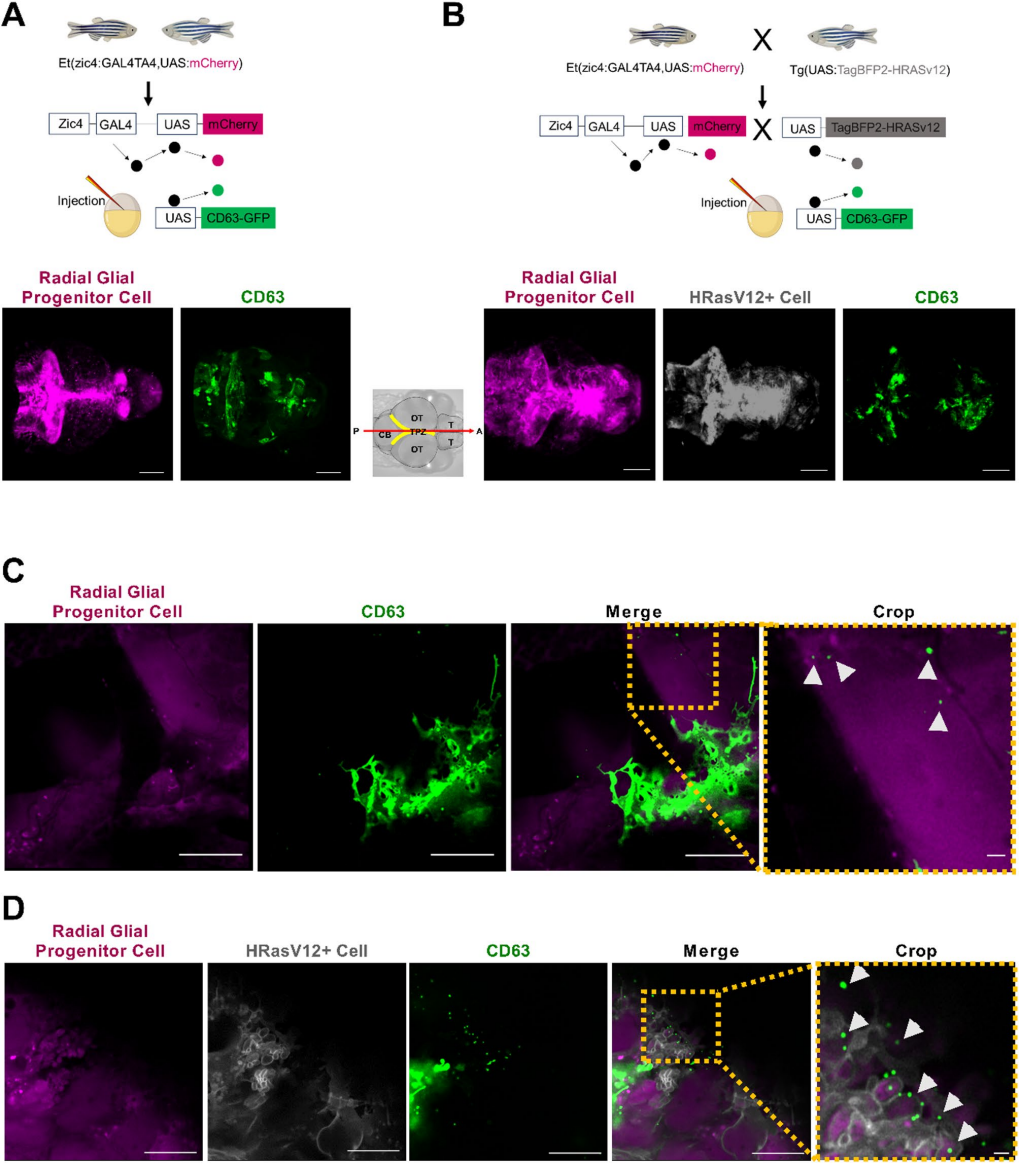
Confocal imaging showed CD63-GFP labelled EVs in HRASv12- and HRASv12 + larval zebrafish brains.** A-B** Schematic representation of the zebrafish injection system used to induce CD63-GFP expression based on the indicated fish lines HRASv12- and HRASv12+. The Zic4 promotor is employed to drive the expression of the transcriptional activator Gal4. Upon binding of Gal4 to the UAS sequence, CD63-GFP expression (under the UAS promoter) is triggered in the HRASv12- line, while HRAS and CD63 are overexpressed in the HRASv12 + line. **A** Confocal images (maximum intensity projections) showing radial glial progenitor cells (magenta) and CD63-GFP + cells (green) in the brain of HRASv12- larvae at 4dpf. Scale bar represents 100 μm. **B** Confocal images showing radial glial progenitor cells (magenta), HRASv12 + cells (grey) and CD63-GFP + cells (green) in the brain of HRASv12 + larvae at 4dpf. Scale bar represents 100 μm. Schematic anterior-posterior dorsal view of the larval brain showing major subdivisions: telencephalon (T), optic tectum (OT), cerebellum (CB) and tectal proliferation zone (TPZ) in yellow. **C** Representative confocal image of radial glial cells (magenta), and released CD63-GFP labelled EVs (arrowheads) in HRASv12- larvae live samples, scale bar 10 μm and crop data scale bar 1 μm. **D** Representative confocal image of radial glial cells (magenta), HRASv12 + cells (grey) and released CD63-GFP labelled EVs (arrowheads) in HRASv12 + larvae live samples, scale bar 10 μm and crop data, scale bar 1 μm

Despite considerable progress in the understanding of the role of EVs in intercellular communication, there is still a lack of a complete understanding of their fate and function in physiological and pathological conditions. To understand their fate and function in vivo, the larval zebrafish model offers outstanding opportunities which enable live imaging of these small particles [17, 22]. Our aim here was to selectively label EVs from a specific cell type mimicking physiological and pathological conditions. To achieve this aim, we focused on labelling EVs released from radial glial progenitor cells and their preneoplastic counterparts using our previously established zebrafish glioblastoma model [24, 38]. This model is based on overexpression of human HRasV12 in radial glial progenitor cells in the developing central nervous system (CNS) [38]. The use of the driver fish line Et(zic4:GAL4TA4,UAS: mCherry)hmz5 [39] results in Gal4-UAS mediated mCherry expression in radial glial progenitor cells (hereafter called HRasV12- larvae) and an outcross of this line to Tg(UAS: TagBFP2-HRasV12) leads to additional expression of TagBFP2-HRASV12 (hereafter referred to as HRASV12 + larvae) inducing a preneoplastic state in these cells [24, 38]. To visualise EVs, the experimental approach was to induce overexpression of CD63 fused to a fluorescent protein (GFP). This was achieved by injecting a pDEST-UAS-CD63-GFP-pA construct into one-cell stage embryos of Et(zic4:GAL4TA4,UAS: mCherry) to achieve expression in HRasV12- larvae (Fig. 7A), while injection into one-cell stage embryos of the outcross to Tg(UAS: TagBFP2-HRasV12) resulted in expression in HRasV12 + larvae (Fig. 7B). This approach allowed us to image EVs released by HRasV12 + and HRasV12- cells in living larvae.

This injection induced a mosaic and transient expression of the CD63-GFP fusion protein in both conditions (Fig. 7A, B). CD63-GFP expression was detected in different regions of the brain including the telencephalon, cerebellum, tectal proliferation zone (TPZ) and optic tectum (OT). Different levels of expression were observed among these regions, with some displaying elevated expression while others exhibited lower levels. Notably, the expression of this construct revealed the presence of EVs in HRasV12- and HRasV12 + larvae (Fig. 7C, D). Moreover, CD63-GFP expression produced a robust GFP signal not only in EVs but also in intracellular compartments, which might be endosomes and lysosomes, as well as at the plasma membrane in both conditions.

**Fig. 8:**
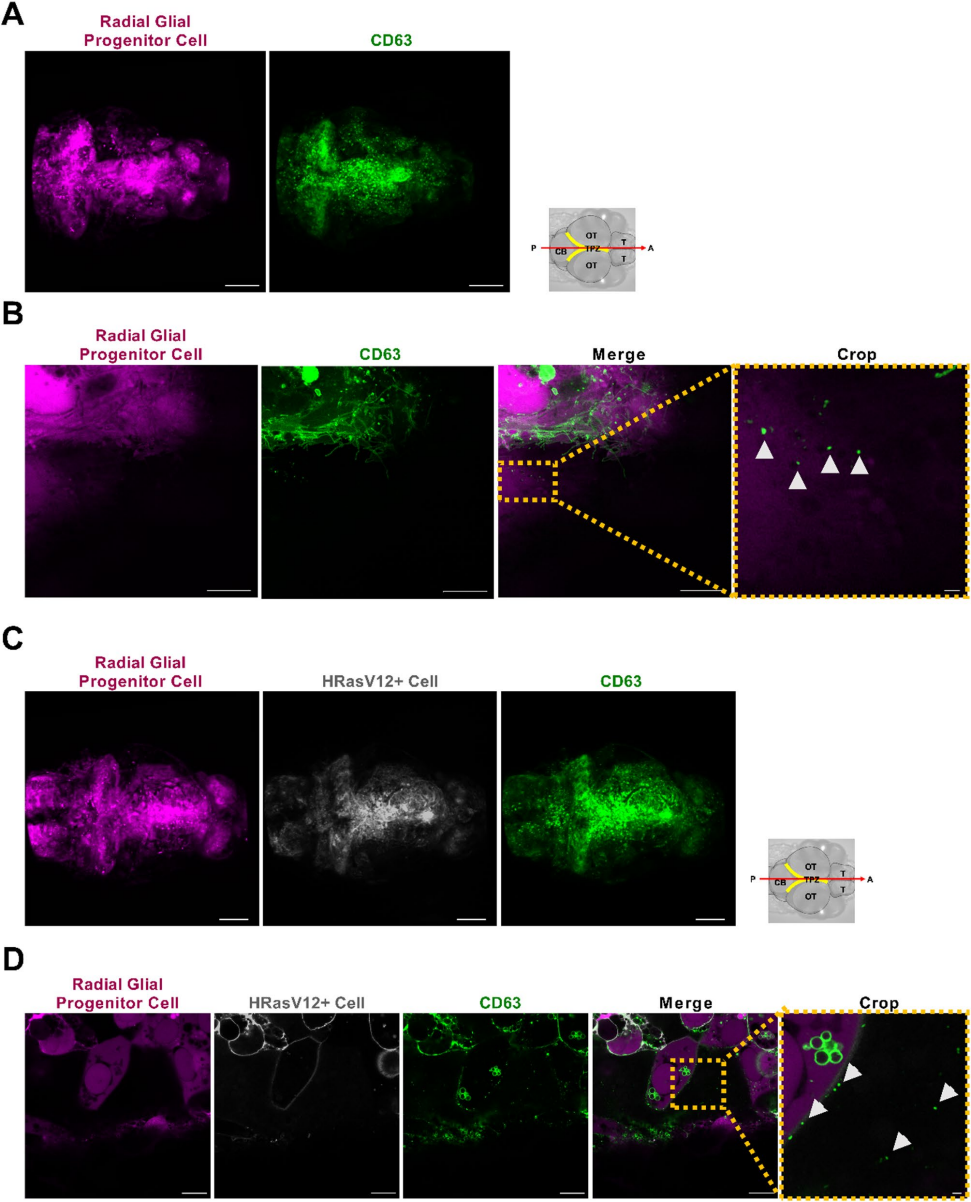
Confocal imaging showed CD63-GFP labelled EVs in HRASv12- and HRASv12 + larval zebrafish brains using the newly generated zic4:GAL4TA4,UAS: mCherry; UAS-CD63-GFP transgenic line.** A** Confocal images (maximum intensity projections) showing radial glial progenitor cells (magenta) and CD63-GFP + cells (green) in the brain of HRASv12- larvae at 4dpf. Scale bar represents 100 μm. **B** Representative confocal image of radial glial cells (magenta) and released CD63-GFP labelled EVs (arrowheads) in HRASv12- larvae live samples, scale bar 10 μm and crop image scale bar 1 μm. **C** Confocal images (maximum intensity projections) showing radial glial progenitor cells (magenta), HRASv12 + cells (grey) and CD63-GFP + cells (green) in the brain of HRASv12 + larvae at 4dpf. Scale bar represents 100 μm. **D** Representative confocal image of radial glial cells (magenta), HRASv12 + cells (grey) and released CD63-GFP labelled EVs (arrowheads) in HRASv12 + larvae live samples, scale bar 10 μm and crop data, scale bar 1 μm

**Fig. 9:**
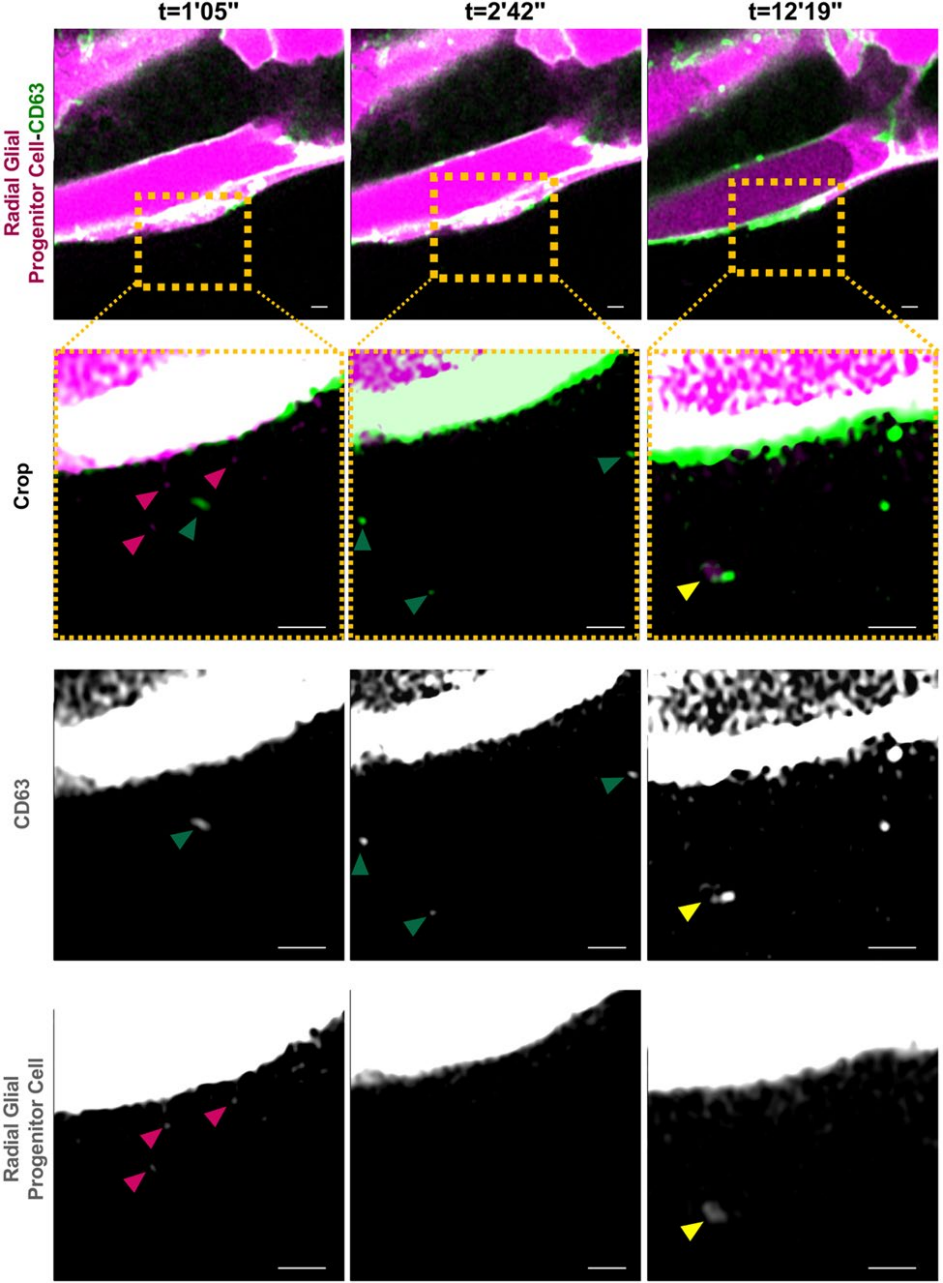
Super-resolution time-lapse imaging revealed EV release by radial glial progenitor cells in the optic tectum of a 4 dpf zic4:GAL4TA4,UAS: mCherry; UAS-CD63-GFP larval zebrafish. Representative confocal images of a time-lapse shown in Additional file 1: Supplemental Video S1 of a radial glial progenitor cell expressing cytoplasmic mCherry and CD63-GFP (top panels – recording times indicated). The dashed box of each time point indicates the area shown at higher magnification below. Depending on time point CD63-GFP + EVs (green arrowheads) and mCherry + EVs (magenta arrowheads) can be detected (left panels). Other time points showed only CD63-GFP + EVs (green arrowheads) (middle panels), or double-positive CD63-GFP+/mCherry + EVs (yellow arrowheads) (right panels). Scale bar represents 1 μm

Finally, to reduce mosaic effects induced by the transient overexpression of CD63-GFP, we generated a stable transgenic line by incorporating the pDEST-UAS-CD63-GFP-pA construct into the Et(zic4:GAL4TA4,UAS: mCherry) line. This new transgenic line zic4:GAL4TA4,UAS: mCherry; UAS-CD63-GFP (hereafter Zic: CD63-GFP) resulted in a strong CD63-GFP signal in HRasV12 + and HRasV12- larvae (Fig. 8A, C). In contrast to the injection strategy, a consistent high expression was observed in all brain regions. Most importantly, in HRasV12 + and HRasV12- larvae the presence of EVs was shown (Fig. 8B, D). By exploiting this newly established fish line, we performed super-resolution timelapse imaging of 4 dpf Zic: CD63-GFP larval OT. We observed the release of three different populations of EVs: CD63-GFP^+^; mCherry^+^ and CD63-GFP^+^/mCherry^+^ (Fig. 9; Additional file 1: Supplemental Video S1).

In conclusion, this strategy allowed us to specifically label EVs from radial glial progenitor cells and their preneoplastic counterparts, using both, a transient injection method as well as a stable transgenic line.

### Detection and characterization of EVs released from radial glial progenitor cells

Using high resolution confocal microscopy, we have shown that we can specifically label EVs in radial glial progenitor cells (HRasV12 + and HRasV12-). Finally, we wanted to test whether these cell-specific EVs can be detected amongst the total EV population isolated from larval heads using our protocol. Hence, EVs were extracted from the Zic: CD63-GFP larvae and NTA was utilised to examine the concentration and size of GFP-tagged EVs.

**Fig. 10:**
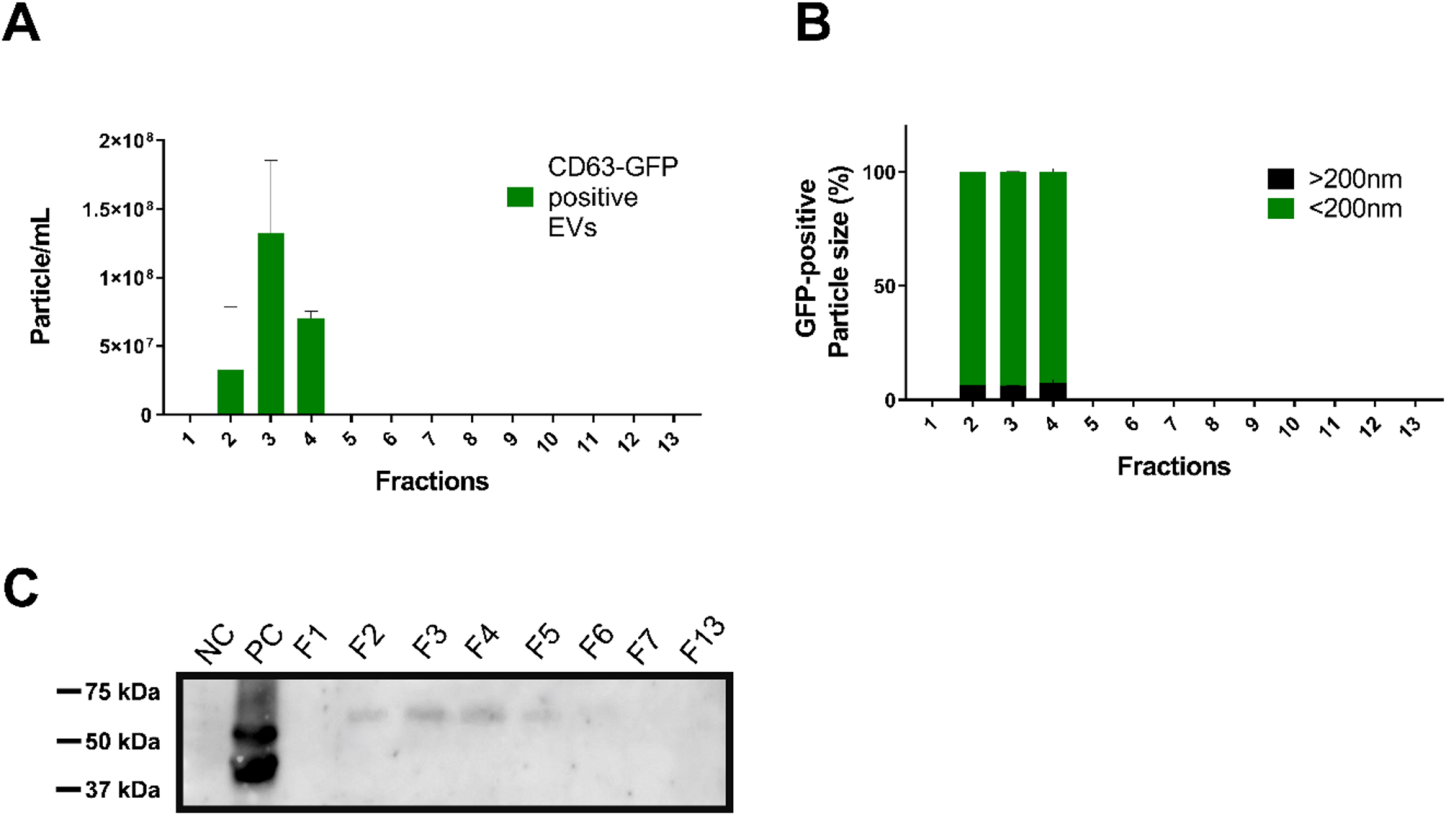
Detection of CD63-GFP positive EVs. EVs were extracted from 4 dpf larval heads of the Zic: CD63-GFP line (*n* = 100 and *N* = 2). **A** Quantification studies of GFP positive EVs using NTA. The average concentrations of CD63-GFP labelled EVs in F2, F3 and F4 were 3 × 10^7^, 1.33 × 10^8^ and 7.5 × 10^7^, respectively. **B** Size analysis of GFP-positive EV. GFP-positive EVs are generally < 200 nm. However, a small proportion, 6.7% ± 0.4% (averaged across all fractions containing GFP-EVs) of particles > 200 nm were detected. **C** Representative western blot analysis showing CD63-GFP (53–62 kDa) in extracted EVs per fraction. Negative Control (NC): water; Positive Control (PC): cellular proteins (eGFP-HRAS: 48 kDa) extracted from Et(zic4:GAL4TA4,UAS: mCherry); Tg(UAS: HRASv12-EGFP) 4 dpf larvae (*n* = 50); F1-F7and F13 fractions: EV proteins. In the positive control derived from the cell lysate of eGFP-HRAS larvae, two bands are detected, which is caused by posttranslational modifications of the HRASV12 protein (prenylated vs. nonprenylated)

NTA analysis revealed that a small number of CD63-GFP labelled EVs were detected within the F2, F3 and F4 fractions (Fig. 10A). The average concentration of CD63-GFP labelled EVs for F2, F3 and F4 were 3 × 10^7^, 1.33 × 10^8^ and 7.5 × 10^7^ particles/mL, respectively (Fig. 10A). These values represent approximately 1–2% of the total amount of EV for each fraction, which is expected considering the fact that these are cell specific EVs within a pool of EVs from all cell types within larval zebrafish heads. Furthermore, we observed that GFP-positive EVs were generally less than 200 nm in size. However, a very small proportion of large EVs were detected in each fraction (average across all fractions 6.7% ± 0.4%) (Fig. 10B). This proportion was similar to the proportion observed in the total amount of EVs.

The presence of CD63-GFP labelled EVs was further confirmed by individual evaluation of each fraction by Western blotting (Fig. 10C). For Western blot analysis, a GFP antibody was utilised to target the CD63-GFP fusion protein. The Western blot results indicated the detection of CD63-GFP labelled EVs throughout the F2 to F6 fraction. Notably, in contrast to the NTA results where CD63-GFP labelled EVs were undetectable in F5 and F6, the Western blot showed greater sensitivity in detecting these EVs.

In conclusion, we showed that our purification method is sensitive enough to detect EVs from specific cell populations in the larval brain.

## Discussion

The role of EVs in intercellular communication has primarily been studied using in vitro cell models and animal or human body fluids. Despite significant progress in understanding EVs as mediators of communication, a comprehensive understanding of their fate and function in intercellular signalling remains incomplete. Hence, the use of animal models is now of great importance to understand the in vivo functionality of EVs. One of the most popular in vivo models in recent years is the zebrafish and elegant studies have demonstrated the advantages of studying EVs in living zebrafish embryos [16, 17, 19, 27, 28]. These studies have already significantly contributed to our understanding of EV release and uptake in vivo. To complement the in vivo imaging, we developed a new EV isolation protocol for larval zebrafish that allows quantification and characterisation of released EVs. While collagenase is a commonly used enzyme for tissue digestion prior to EV isolation, it can have a detrimental impact depending on the needs of downstream analyses. Recently, Matamaros-Angles and colleagues showed for example that collagenase treatment results in cleavage of EV membrane proteins, while intraluminal proteins and nucleic acids were not affected [40]. Intriguingly, effects of collagenase on cell viability have not been shown before. Any impairment to cell viability during this phase could significantly impact subsequent investigations, especially when compounds are used that interfere with EV release. Ruptured cellular membranes might result in the release of EVs in these samples, which hinders accurate quantification of the amount of released EVs. Hence, our initial focus was on assessing the impact of collagenase treatment, manual homogenization and *Bacillus Licheniformis* protease enzyme treatment on cell viability during the tissue dissociation stage. Our results indicated that collagenase had a significant impact on cell viability (35% ± 1.6% cell death). Manual homogenization led to a slightly better result (12% ± 0.5% cell death), which is in line with previous data showing that the rate of dead cells is highly variable upon manual homogenization and can be up to 26.7% [25]. Importantly, the *Bacillus Licheniformis* protease enzyme exhibited the most favourable outcomes, exerting minimal impact on cell viability (4% ± 1.3% cell death). Hence, we consider this enzyme most suitable for tissue digestion to extract EVs. Of importance is also the temperature difference between Collagenase protocols and the Bacillus licheniformis protocol. Collagenase protocols typically require higher temperatures (often around 37 °C) to achieve efficient tissue dissociation. However, these elevated temperatures can induce cellular stress, alter gene expression profiles, and reduce cell viability, potentially affecting data interpretation. In contrast, Bacillus licheniformis digest is effective at lower temperatures (4 °C), which helps preserve cell viability and phenotype by minimizing heat-induced stress and proteolytic damage. This improved preservation is crucial for obtaining accurate molecular profiles in downstream applications such as single-cell sequencing and multiomics.

Although UC and DGUC are commonly used for EV isolation, they have certain limitations. UC yields high levels of EVs, but these are often contaminated with protein aggregates. This technique may affect the biological functionality of EVs due to the high centrifugal forces to which the samples are subjected [29]. Conversely, DGUC is reported to have a lower EV recovery rate with a higher purity, nevertheless the use of different density gradient compounds might impact on the composition of the protein corona of EVs [30]. Our protocol provides a new, robust and reproducible approach for the extraction of EVs from larval zebrafish using SEC to achieve a better yield than UC. Moreover, SEC has proven to be a promising, adaptable method for high-throughput EV isolation from various biofluids like plasma, urine, and cell culture medium [41–44]. A previous study has demonstrated the preservation of biophysical and functional EV properties using SEC compared to DGUC [31]. Nonetheless, this method has not previously been applied to zebrafish larvae. Hence, we evaluated every individual step crucial to the efficiency and suitability of this method one by one in the context of its application to zebrafish larval heads.

A crucial factor in the SEC application involves identifying the most suitable fractions for EV collection, which varies based on the source material (such as cell culture medium, plasma, urine, etc.) and the specific sample preparation approach. At this point, 4 different strategies of sample preparation were tested which also included the combination of UF. Combinations of SEC with UF have been used in the literature for different EV sources [44–46], including studies showing that this combination strategy increases sample throughput, scalability and selectivity [44, 45, 47]. Eventually, based on the distribution of the fractions, contamination of proteins and TEM analysis, we concluded that the most appropriate sample preparation strategy to extract EVs from cells and debris from larval zebrafish heads was 10,000 g + UF. Additionally, we found that utilising 50 zebrafish larval heads provided optimal results for this protocol, yielding a higher particle count compared to using 25 larvae. Conversely, employing 100 zebrafish larval heads resulted in the highest particle count including appearance of particles in later fractions (F7-F11). This observation could be explained by partial saturation of the column caused by the high concentration of particles, tissue debris and proteins in the sample. This reduces the column’s ability to effectively separate particles based on their size. As a result, particles that would typically appear in earlier fractions (F2-F5) may be delayed and instead elute in later fractions (F7-F11). However, these later fractions show protein contaminants and hence might not be suitable for downstream applications.

Several studies have employed different approaches to label EVs in zebrafish. Verweij et al. for example labelled EVs using the human *CD63* sequence generating a pUbi-CD63-pHluorin plasmid while Scott et al. used membrane-tethered fluorophores to label EVs [17, 19]. The findings of Verweij et al. (2019), provided robust in vivo evidence demonstrating that CD63-pHluorin–positive structures predominantly correspond to exosomes. Through live imaging, they demonstrated that CD63-pHluorin labels vesicles that are trafficked via the MVB pathway and subsequently released into the extracellular space [17]. The expression strategy described here allows the specific identification of EVs released from radial glial progenitor cells and their preneoplastic counterparts due the combination of a cell specific promoter and the Gal4-UAS system. Based on the existence of a large number of cell specific Gal4 driver lines in different species (e.g. zebrafish, fruit fly), this offers high versatility to specifically visualise EVs from almost every cell type of interest. The zebrafish CD63-GFP fusion protein employed here facilitated the visualisation of EVs, as well as allowed the labelling of additional cellular structures, such as the plasma membrane and MVB due to the localisation of CD63. The use of pH-sensitive fluorescent proteins (phuji or phluorin) here would provide the advantage of reducing the cellular background from the plasma membrane and MVBs and allow specific visualisation of released EVs [17, 48]. Interestingly, our super-resolution live imaging in the optic tectum of *zic4:GAL4TA4*,*UAS: mCherry; UAS-CD63-GFP* transgenic zebrafish revealed the release of different EV populations. We observed the release of three different populations of EVs: CD63-GFP^+^; mCherry^+^ and CD63-GFP^+^/mCherry^+^. We speculate these populations represent different types of vesicles with distinct origins. The CD63-GFP^+^ vesicles would likely originate from the exosome pathway secreted from MVBs. However, the mCherry^+^ and CD63-GFP^+^/mCherry^+^ ones could respectively be small and large EVs generated from the ectosome pathway, explaining the presence of cytosolic molecules and the difference of size. In light of these findings and given the conserved role of CD63 as an sEVs marker across model systems, we consider it a well-supported and widely accepted interpretation that the CD63-GFP⁺ puncta observed in our experiments correspond to EVs.

In this study, we demonstrate for the first time that GW4869, an inhibitor of ESCRT-independent EV biogenesis, effectively blocks EV generation in a dose-dependent manner in larval zebrafish, with no significant changes observed in EV particle size distribution. GW4869, a selective nSMase2 inhibitor, is one of the most widely used pharmacological agents to inhibit EV formation and EV release. Numerous studies have confirmed the efficacy of GW4869 in inhibiting EV release across various in vitro cell lines [49–52], For example, McNamee et al. (2023) reported EV inhibition in triple-negative breast cancer cells highlighting that GW4869 was associated with an apparent increase in particle sizes in these models [52]. Menck et al. (2017) observed a reduction in EV release in SKBR3 human breast cancer cells and mouse L-cells [49]. Furthermore, Simon et al. (2018) emphasised the impact of GW4869 on the glioblastoma cell line (U87), although they did not report similar findings for the LN18 cell line [50]. All these studies, including our own, used NTA systems to evaluate EV concentration and size after GW4869 treatment. A prior study investigated the effect of GW4869 on EV release in larval zebrafish, reporting no significant impact on EV release following treatment [17]. However, the authors monitored EV release from the yolk syncytial layer (YSL) in zebrafish, suggesting that EV biogenesis might be different in the YSL compared to the larval head. We demonstrate a clear dose-dependent effect of GW4869 on EV inhibition in larval zebrafish heads, a finding that underscores the importance of dosing and treatment duration, which were not detailed in prior research. Our findings contribute to the expanding evidence that supports GW4869 as an effective tool for in vivo EV inhibition and highlights the sensitivity of our newly developed EV isolation protocol.

## Conclusion

In this study, we present an optimized protocol for the isolation and characterization of EVs from larval zebrafish tissues, enabling accurate quantification of released EVs without compromising cell viability. This advancement facilitates reliable comparison between treatment conditions and strengthens the utility of zebrafish as a model for in vivo EV research. Additionally, we introduce a cell-type-specific EV labelling strategy using the UAS: CD63-GFP system, which enhances the capacity to study EV origin and dynamics within intact tissues.

Despite these advances, the study has certain limitations. While our enzymatic tissue dissociation methods were carefully evaluated for their impact on cell viability, comprehensive assessment of potential effects on EV surface proteins remains limited. This is primarily due to the current lack of well-validated antibodies against zebrafish-specific EV membrane markers. As antibody resources expand, future studies will be able to more fully evaluate the preservation of EV surface integrity. In addition, while the UAS: CD63-GFP system enables cell-type-specific EV visualization, background fluorescence from membrane-associated structures presents a challenge to signal specificity. Further refinement of reporter constructs, or imaging strategies may enhance resolution and reduce non-specific signals. Finally, although the Gal4/UAS system offers exceptional flexibility for targeted expression in zebrafish and *Drosophila*, its use in mammalian models is more limited. Nonetheless, the principles established here lay the groundwork for the development of parallel tools in other organisms, paving the way for broader translational application.

## Supplementary Information

Supplementary Material 1

Supplementary Material 2. 

## Data Availability

The data underlying the current study are available from the corresponding author on reasonable request.

## Abbreviations

BSA: Bovine serum albumin
DGUC: Density gradient ultracentrifugation
dpf: Days post-fertilisation
EVs: Extracellular vesicles
NTA: Nanoparticle tracking analysis
OT: Optic tectum
SEC: Size exclusion chromatography
TEM: Transmission electron microscopy
TPZ: Tectal proliferation zone
UC: Ultracentrifugation
UF: Ultrafiltration
YSL: Yolk syncytial layer
