# Supplementary material for: Enabling mechanistic studies of EVs in vivo: a protocol for isolation and cell-specific labelling in larval zebrafish
Source: Cell Commun Signal. 2025 Oct 14;23:436. doi: 10.1186/s12964-025-02433-3 (PMC12522632; doi:10.1186/s12964-025-02433-3)
Supplement: Supplementary file 1 — Supplementary Material 1 [file 12964_2025_2433_MOESM1_ESM.docx]

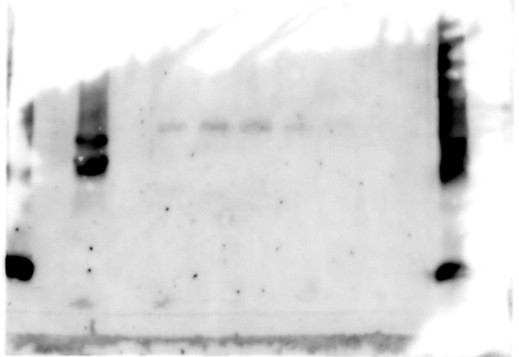


M NC PC F1 F2 F3 F4 F5 F6 F7 F13 M

**Supplementary Figure 1. Detection of CD63-GFP positive EVs by western blot (uncropped data)**

Representative uncropped western blot image showing detection of CD63-GFP (expected size: 53–62 kDa) in EV protein samples across various fractions. Negative Control (NC): nuclease-free water. Positive Control (PC): cellular protein lysate containing eGFP-HRAS (48 kDa), derived from whole larvae of *Et(zic4:GAL4TA4,UAS:mCherry); Tg(UAS:HRASv12-EGFP)* at 4dpf (n=50). EV protein samples correspond to fractions F1–F7 and F13. Protein cleavage of eGFP-HRAS was observed in the PC (cell lysate), while no such cleavage was detected in CD63-GFP positive EV fractions. This confirms the integrity of EV-associated CD63-GFP in isolated vesicles.
